# Do Hospital Leaders Live in the Communities They Serve? A Comparative Analysis

**DOI:** 10.1089/heq.2021.0147

**Published:** 2022-04-21

**Authors:** Charles Sanky, Hannah Johnson, Daniel Sanky, Jacob M. Appel

**Affiliations:** ^1^ Department of Medical Education, Icahn School of Medicine at Mount Sinai, New York, New York, USA.; ^2^ Department of Emergency Medicine, Icahn School of Medicine at Mount Sinai, New York, New York, USA.; ^3^ Graduate Program in Public Health, Icahn School of Medicine at Mount Sinai, New York, New York, USA.; ^4^ Graduate School of Medical Sciences, Weill Cornell Medicine, New York, New York, USA.; ^5^ Department of Psychiatry, Icahn School of Medicine at Mount Sinai, New York, New York, USA.

**Keywords:** health equity, community health, health system leadership, medical sociology, demographics, race

## Abstract

**Purpose:**

Many factors contribute to persistent intractable disparities in health care, but the geographic separation of health care executives and patient communities has not been explored. From Congresspeople to police officers, individuals engaged in public service often face criticism for not living in the neighborhoods where they work. These critiques stem from the belief that to engage meaningfully with a community, one has to understand its experiences and share its interests—and geographic proximity offers one opportunity to bridge such divides. This article seeks to determine whether the senior executive leadership of American hospitals live in the same communities as their patient populations.

**Methods:**

From August 2020 to January 2021, the research team identified the leadership of the “largest” and “best” hospitals in the United States (*n*=68). Public directories were used to locate residential addresses. Newly released U.S. Census data provided proportions of individuals identifying as black/African American and Hispanic/Latinx in each zip code. Respective demographic proportions of hospital communities and hospital leadership residence were compared.

**Results:**

Hospitals shared the same zip codes with only three health system leaders (4.41%), seven hospital leaders (10.45%), and six deans (10.91%) of respective institutions. Hospital leadership lived in zip codes with a significantly lower proportion of black/African American (*p*<0.0009) and Hispanic/Latinx (*p*<0.0036) residents than their hospital communities.

**Conclusion:**

This article reveals significant differences between where health care leaders live and where they work. Future research should investigate the impact of residential disparities and the consequences of potential remedies on health equity.

## Introduction

A consensus has emerged over the past 50 years that social determinants play a significant and often determinative role in medical outcomes.^1^ One of the rare positive developments of 2020 was an increased focus on health care inequities.^2^ This renewed attention stemmed from two phenomena that drew widespread attention to systemic racism: The disparate impact of the COVID-19 pandemic on communities of color and the tragic killing of George Floyd that reinvigorated Black Lives Matter protests. In the wake of Floyd's death, numerous hospitals and medical schools issued statements condemning racism and committing to strive for equity, as did the American Hospital Association,^3^ the American Association of Medical Colleges,^4^ and the National Medical Association.^5^ Yet, these pledges occurred against a backdrop of limited progress.

Health care disparities between people identifying as white and those identifying as African Americans have not ameliorated significantly over the past 70 years, and in some areas—such as differences in the rates of heart disease—they have actually worsened considerably.^6^ The life expectancy gap between whites and blacks/African Americans has decreased during this period, but whites on average still live 4 years longer.^7^ African Americans also remain underrepresented in health care leadership positions.^8^ While some progress has been achieved in diversifying the medical workforce, the number of black men in medical school at present remains lower than in 1978.^9^ Similarly, Hispanic Americans continue to receive less preventive care than white Americans.^10,11^

Many factors contribute to these persistent and intractable disparities. One potential factor that has not previously been explored in the literature is the geographic separation of health care executives and the communities that they serve. From Congresspeople to police officers, individuals engaged in public service often face criticism for not living in the neighborhoods where they work.^12–14^

These critiques stem from the belief that to engage meaningfully with a community, one has to understand its experiences and share its interests—and geographic proximity offers one opportunity to bridge such divides. Considerably less attention, if any, has been devoted to these concerns in health care, despite an emergent need to reconcile the clear health disparities that affect historically marginalized populations. If hospital leadership were able to better understand the lived experiences of the communities they serve, perhaps health care systems could rebuild a lost sense of trust, close gaps in clinical care quality, and improve health outcomes equitably for all patients.

This article seeks to answer a relatively straightforward question: Do American health care leaders live in the same communities as their patient populations? Or at least, do they live in communities whose racial makeups resemble those of the communities that their hospitals serve?

## Methods

Our research team consisted of medical students, graduate students, and faculty with experience in quantitative and qualitative research methods. The team completed hospital selection, data collection, demographic data review, and statistical analysis from August 2020 to January 2021. This observational study of publicly accessible data was submitted to the Icahn School of Medicine at Mount Sinai Institutional Review Board and deemed exempt from review.

### Hospital selection

We selected hospitals for review based on size and rankings. A list of the 60 largest hospitals in the United States, ranked by number of inpatient hospital beds, is reviewed annually by *Becker's Hospital Review*.^15^ The number of beds listed in this review was independently verified by the study team using each hospital's website and reported data. We included these 60 hospitals for review, recognizing that their high level of throughput likely signifies a large patient population served. In addition, the “best hospitals” in the United States are selected by the annual *U.S. News & World Report* rankings.

This list remains a gold standard utilized by health care leaders and consumers alike, reflecting the highest quality health institutions. In 2020, 21 hospitals were ranked as the best hospitals in the United States,^16^ 13 of which were also included in the *Becker's Hospital Review*. Sixty-eight hospitals were considered for review in this study (Table 1). The study investigators created a shared spreadsheet, populated with the hospital name, number of beds, and hospital zip code from the aforementioned lists.

**Table 1.** Best and Largest Hospitals, Per U.S. News and World Report and Becker's Hospital Review (by Number of Inpatient Hospital Beds, Descending)

Hospital name	City	State	Hospital beds
Barnes-Jewish Hospital^a^	St. Louis	MO	1638
Yale New Haven Hospital^a^	New Haven	CT	1541
Ohio State University Wexner Medical Center^a^	Columbus	OH	1509
Jackson Memorial Hospital^a^	Miami	FL	1500
AdventHealth Orlando^a^	Orlando	FL	1364
Cleveland Clinic^a^	Cleveland	OH	1285
Mayo Clinic Hospital-Saint Mary's Campus^a^	Rochester	MN	1265
Atrium Health Carolinas Medical Center^a^	Charlotte	NC	1211
Memorial Hermann-Texas Medical Center^a^	Houston	TX	1182
The Johns Hopkins Hospital^a^	Baltimore	MD	1162
UAB Hospital^a^	Birmingham	AL	1157
Mount Sinai Hospital^a^	New York	NY	1134
Beaumont Hospital—Royal Oak^a^	Royal Oak	MI	1131
Saint Francis Hospital	Tulsa	OK	1112
Miami Valley Hospital^a^	Dayton	OH	1101
UF Health Shands Hospital^a^	Gainesville	FL	1095
Baylor University Medical Center^a^	Dallas	TX	1042
Massachusetts General Hospital^a^	Boston	MA	1042
Tampa General Hospital^a,b^	Tampa	FL	1006
Vanderbilt University Hospital^a^	Nashville	TN	1000
Vidant Medical Center^a^	Greenville	NC	974
Huntsville Hospital	Huntsville	AL	971
Duke University Hospital^a^	Durham	NC	957
Houston Methodist Hospital^a^	Houston	TX	952
UCSF Medical Center^a^	San Francisco	CA	950
Orlando Regional Medical Center	Orlando	FL	941
Inova Fairfax Hospital^a^	Falls Church	VA	923
MedStar Washington Hospital Center^a^	Washington	DC	912
Thomas Jefferson University Hospital^a^	Philadelphia	PA	908
Christiana Hospital^a^	Newark	DE	906
Medical City Dallas^b^	Dallas	TX	899
Northwestern Memorial Hospital^a^	Chicago	IL	894
Cedars-Sinai Medical Center	Los Angeles	CA	886
Wake Forest Baptist Medical Center^a^	Winston-Salem	NC	885
Parkland Health and Hospital System^a^	Dallas	TX	882
Hartford Hospital^a^	Hartford	CT	867
Lakeland Regional Health Medical Center^a^	Lakeland	FL	864
NYP Weill Cornell Medical Center^a^	New York	NY	862
Mercy Hospital St. Louis	St. Louis	MO	859
Novant Health Forsyth Medical Center	Winston-Salem	NC	859
Aurora St. Luke's Medical Center of Aurora Health Care	Milwaukee	WI	854
Texas Heart Institute at Baylor St. Luke's Medical Center^a^	Houston	TX	850
NYU Langone Hospitals^a^	New York	NY	844
Baptist Health—Little Rock	Little Rock	AR	843
Montefiore Hospital—Moses Campus^a^	Bronx	NY	816
Methodist Hospital^b^	San Antonio	TX	811
University of Maryland Medical Center^a^	Baltimore	MD	806
UNC Medical Center^a^	Chapel Hill	NC	803
Mayo Clinic Hospital—Methodist Campus^a^	Rochester	MN	794
ProMedica Toledo Hospital^a^	Toledo	OH	794
Brigham and Women's Hospital^a^	Boston	MA	793
Lehigh Valley Hospital—Cedar Crest	Allentown	PA	780
Penn Presbyterian Medical Center^a^	Philadelphia	PA	776
Ochsner Medical Center^a^	Jefferson	LA	775
UMass Memorial Medical Center—Memorial Campus^a^	Worcester	MA	773
Hackensack University Medical Center^a^	Hackensack	NJ	771
Albany Medical Center^a^	Albany	NY	766
The University of Kansas Hospital^a^	Kansas City	KS	750
NYP Columbia University Irving Medical Center^a^	New York	NY	738
North Shore University Hospital^a^	Manhasset	NY	738
Reading Hospital^a^	West Reading	PA	735
Rush University Medical Center^a^	Chicago	IL	727
Broward Health Medical Center^a^	Fort Lauderdale	FL	716
Stanford Hospital^a^	Stanford	CA	605
Michigan Medicine—University Hospital^a^	Ann Arbor	MI	550
UCLA Medical Center^a^	Los Angeles	CA	520
Keck Hospital of USC^a^	Los Angeles	CA	401
Mayo Clinic-Phoenix^a^	Phoenix	AZ	268

^a^ Indicates hospitals with an academic medical or graduate health school affiliate.

^b^ Indicates hospitals that are considered part of a for-profit health system.

### Data collection

The research team searched each hospital's website to collect and record information regarding the name of the health system of which it is a part, nonprofit/for-profit status, health system president/Chief Executive Officer (CEO) name, and hospital president/CEO name. If the hospital was affiliated with a medical or graduate school, the name of the institution's dean or president was also recorded through searching school websites for leadership and administration. Our approach to hospital leadership represented the traditional paradigm of there being an individual president/CEO of the hospital, a president/CEO of a health system leader, and a dean or president of academic affairs for a related medical school.

To protect privacy, zip codes were the only element from the address field recorded in our data collection process. For each person listed, the individual's full name was entered into fastpeoplesearch.com. This directory was chosen for the primary method of data collection, as it appeared to be the most up-to-date directory as determined during methodology pilot testing. Full names were included without credentials, and searches were conducted in the same state as the hospital location.

Formal names, nicknames, prior employment, age, and spouse were used to refine searches. They were uncovered through review of biographies available on health system websites and newspaper publications. Names were only considered a match if (1) only one match resulted and there were no indicators that it might be incorrect, or (2) there were matches by name and two additional parameters, such as age and spouse. As multiple locations were frequently listed, other websites such as whitepages.com with publicly accessible information were utilized. Ultimately, zip codes associated with addresses most proximal to the hospital or health system headquarters were selected for inclusion to exclude vacation, summer, or other homes.

Stringent criteria and double entry methods were utilized to ensure data validity. Individual members of the research team reviewed each hospital leader and provided a zip code independently. Only when a zip code was confirmed by both individuals was the entry considered valid. Discrepancies were addressed through investigation and consensus by the study's senior authors.

Due to their size, some hospitals have entire zip codes devoted solely to their hospital centers, or even allocated to portions of their centers. In a review of the 68 hospitals, 8 had their own zip codes. No population demographic information exists for such zip codes, as census data are collected from populations residing in each zip code. To correct for this, the research team selected alternate zip codes based on the bordering or closest zip code from the hospital's emergency department, as the emergency department often serves as the public's front door to the hospital.

Retrospectively, after collecting zip codes for health leadership, zip codes were mapped relative to the hospitals. If any health care leader's residential zip code was listed within a 15-min drive from the hospital, that zip code was considered the alternate zip code. This method conservatively designated as many hospital leaders as living in their hospital community as possible in this particular case.

### Demographic data review

The American Community Survey (ACS) is an annual survey conducted by the U.S. Census Bureau.^17^ Spanning demographics, housing, jobs, education, and other information, the ACS is considered the gold standard in population data. The 5-year ACS estimates represent data collected over time and therefore confer increased statistical reliability, despite a population's size and subgroup composition.^18^ As such, ACS 5-year estimates are thought to provide both the most reliable data on demographic composition at the zip code level of geographic granularity.

At present, the 2015–2019 ACS 5-Year Estimate Data are considered the most up-to-date, publicly available data set for populations in the United States.^19^ Released on December 10, 2020, this period estimate was utilized in our analysis. The 2015–2019 ACS 5-Year Estimate Data were queried for race and ethnicity and downloaded as a Comma Separated Value (CSV) file for analysis, organized by zip code. Percentages were calculated for number of individuals per zip code self-identifying as black, African American, Hispanic, or Latinx—categories representing populations typically underserved in medicine.^20^

Using the XLOOKUP command in Microsoft Excel, the research team queried the 2015–2019 ACS 5-Year Estimate data set to reference each zip code, returning the percentages of black/African American and Hispanic/Latinx populations of each zip code collected (hospital zip code, health system leader zip code, hospital leader zip code, and dean zip code).

### Statistical analysis

The research team conducted descriptive analyses to examine the distribution, central tendencies, normality, and other characteristics of the data. Aggregate percentages of all hospitals for black/African American and Hispanic/Latinx populations were characterized, as well as aggregate percentages for each cohort of health system president, hospital presidents, and dean's residential zip codes. The research team compared percentages for the black/African American and Hispanic/Latinx populations for each zip code collected. After testing for normality, *t*-tests and Mann–Whitney *U*-tests were conducted where appropriate. All analyses were performed using Microsoft Excel and SAS 9.4.^21^

## Results

Of the 68 hospitals considered for this analysis, the research team identified names and zip codes of residence for 68 health system leaders (100%) and 67 hospital leaders (98.53%). Fifty-seven of these hospitals (83.82%) had an academic medical or graduate health school affiliate, and names and zip codes were identified for 55 of their deans (96.49% of affiliated medical schools, 80.88% of total hospitals reviewed). While most were considered nonprofit hospitals and health systems, three (4.41%) were considered for-profit institutions.

To protect health leader privacy, individual hospital leaders' zip codes are not reported in this analysis; however, aggregate results are explored. Hospitals shared the same zip codes with only three health system leaders (4.41%), seven hospital leaders (10.45%), and six deans (10.91%) of their respective institutions.

As there were outlier values for communities and hospital leaders, median values were utilized to better characterize the central tendency of data. Candidate hospitals had populations identifying as black or African American (median=12.60%) and Hispanic or Latinx (median=10.15%) in their neighborhoods. Table 2 shows the median percentages of these population groups across zip codes for each level of hospital leadership, as well as significance compared with the hospital zip code's demographics.

**Table 2.** Black/African American and Hispanic/Latinx Populations in Hospital and Leadership Zip Codes

	Hospital	Health system president	Hospital president	Dean
Black/African American (median % of population)	12.60	5.25^**^	5.40^**^	5.20^**^
Hispanic/Latinx (median % of population)	10.15	6.35^*^	6.20^*^	6.30^**^

^*^ Significant at *p*<0.05 in comparison with population percentage of hospital zip code in the respective row.

^**^ Significant at *p*<0.001 in comparison with population percentage of hospital zip code in the respective row.

Supplementary Figures S1 and S2 show the distributions of hospitals and each cohort of hospital leadership, for black/African American and Hispanic/Latinx populations, respectively. These data illustrate the gaps in racial and ethnic composition between senior executives' neighborhoods and the neighborhoods their hospitals serve.

Each cohort failed to approximate a normal distribution, and so, medians and Mann–Whitney *U*-tests were utilized as a nonparametric approach to measure differences between each cohort and hospitals' zip code demographic composition. Health system presidents, hospital presidents, and deans all lived in zip codes with a significantly lower proportion of residents identifying as black or African American (*p*<0.0001 to *p*<0.0009) and a significantly lower proportion of residents identifying as Hispanic or Latinx (*p*<0.0005 to *p*<0.0036) than their hospital communities.

## Discussion

The top-line result is that senior health care executives live in communities that are fundamentally different in racial and ethnic character from the communities in which their institutions are situated. These differences are profound. They may reflect economic differences between high-earning health care executives and communities of color, who consistently earn less than white Americans.^22^ Differences may also reflect a desire, either conscious or unconscious, of senior health care executives to live among people who “look like” themselves.

Deans of medical schools are more likely to live in the communities in which they work than health system leaders (10.91% vs. 4.41%) and have higher percentages of minority populations in their zip codes compared with other hospital leadership. This may reflect lower salaries for deans when compared with health care executives, more diversity among the former, or a difference in the political and social values of those individuals who lead medical schools versus those who choose to lead hospital systems. In addition, these differences may reflect the growing trend of multiple hospital campuses consolidating into a larger health system, headed by a single CEO.

All hospitals included in this analysis are considered system affiliated; none is a stand-alone hospital. In practice, most of these represent the flagship hospital in a network of hospitals, or the only hospital with many smaller physician practices and clinics as a system. This growing trend reflects how the current health care landscape has required hospitals to be a part of larger health systems. Even hospitals frequently perceived as stand-alone, such as community hospitals and county hospitals, are legally, financially, and operationally considered parts of health systems for this same reason.

The hospitals selected in this analysis tend to represent the highest volume hospitals with high number of beds and a number of patients served, which does overrepresent hospitals as parts of systems. Nevertheless, the fact that they remain a part of a larger system is precisely the reason the leadership of the hospital, health system, and medical school were analyzed separately.

Distributions of each cohort (hospital, health system president, hospital president, and dean) were asymmetrical and did not pass the tests of normality, necessary for traditional *t*-tests. As such, two-tailed Mann–Whitney *U*-tests were used despite lower power to measure differences between the demographics of hospital zip codes and each cohort of hospital leadership.

In such analysis, we conservatively chose to report the *p*-values for t approximation, which were all substantially higher than the *p*-values for the Z statistic. This test, which relies on median instead of mean, underestimates the degree of difference afforded by the 11 hospital zip codes, which were considered outliers, having substantially higher proportions of black, African American, Hispanic, and Latinx populations compared with the other hospitals. Without this adjustment, our findings would suggest even greater discrepancies in demographics between where health care leaders and patients live.

Most of the “best” and “largest” hospitals in the United States function as nonprofits. Our analyses between for-profit and nonprofit hospitals found no statistically significant differences among groups; however, the limited sample size (three hospitals) in the for-profit category does not lend the statistical power for adequately making such a comparison. Despite the resulting exemption from federal and state taxes, and often local property assessments, an institution's nonprofit status is supposed to reflect its willingness to use its resources to further the welfare of the local communities.^23^ An increasing body of evidence suggests that the financial exemptions afforded to many nonprofits are not justified by the economic benefits they claim to provide to their communities.^24^

Senior health care executives who receive extraordinary levels of compensation, which could otherwise aid the hospital's local community, may instead spend those funds upon residential property taxes in other wealthier communities. Such circumstances raise serious questions about the cost–benefit calculus of a hospital's nonprofit status. As our data suggest, this draining of resources is concerning due to the disparate impact upon racial and ethnic minority groups. No concomitant benefit of having health care leaders live in separate communities from their hospitals appears to outweigh the sapping of resources.

In addition to these direct economic effects, the intangible consequences of such self-segregation are deeply problematic. How are health care leaders to recognize and relate to the challenges of the individuals whom their institutions serve when they travel in far-removed social and economic orbits? If hospital leaders do not live among the communities which their institutions are meant to serve, what other measures are they taking to understand the experiences and interests of local patient populations?

### Limitations

This study has several limitations that warrant consideration. The 68 hospitals included in this study are considered largest based on numbers of beds and “best” due to rankings. While hospitals were selected to reflect large patient populations and patients often requiring more advanced levels of care, our findings do not necessarily characterize all hospitals and are inherently limited by a small sample size. Many of the institutions in these lists, due to the nature of their size or national recognition, devote substantial funding to conducting nationwide searches and attracting leadership talent.

This phenomenon further underscores our findings, and future analysis should investigate the extent to which “outsiders” are brought in to lead health institutions in communities of which they have no ties, and what, if any, measures these leaders take to understand the local community.

Nevertheless, there are many other hospitals in the United States, including local and community institutions, which do not necessarily have this experience. It is possible that senior health care executives may have derived from physicians or administrators with long-standing ties to their local hospital and community, although further exploration is required to substantiate this claim. Alternatively, these findings may be further exaggerated at for-profit hospitals, which are underrepresented in our sample.

Despite robust methodology for data collection through stringent criteria, double entry, and reconciling conflicting data, zip code data for hospital leadership were not solicited from individuals directly. Our approach hinged upon publicly accessible records for local addresses of health leaders, which may not always be valid or complete. Efforts to validate the data, such as triangulation of locations using multiple web search engines, multiple family members, and published biographical information and new stories, were conducted. Still, these public records may not reflect the reality of health leadership residence. Some leaders had multiple addresses of residence listed; the zip code considered closest to the hospital was conservatively utilized for the purpose of this study.

It is possible that a hospital president might, for example, have a home in a different town but rent an apartment a block away from the hospital and thus spend all of the time in the immediate community in which the hospital is located. While this reconciles some concerns about understanding and living in the community, the fact cannot be dismissed that the hypothetical leader calls another place home.

This analysis focused on historically disadvantaged communities in medicine, specifically including individuals self-identifying as black, African American, Hispanic, or Latinx. There are other populations that are considered medically underserved, including entire geographic regions that have limited access to health care services, high infant mortality, high poverty, too few primary care providers, or a substantial elderly population.^25^ While not included here, further analysis should reflect these other disadvantaged groups.

Although hospital service areas usually represent larger regions spanning numerous zip codes, we chose to focus on areas deemed in the immediate vicinity or metaphorical backyard of these institutions, as they are most impacted by this trend. Some hospitals function as quaternary referral centers and may draw from a catchment area far outside of their immediate zip codes.

One final limitation concerns the mere effect of a hospital's presence in a community on the community itself. Gentrification refers to the process by which the character of a neighborhood changes through the influx of more affluent residents and businesses.^26^ Rent and property values increase, often at the cost of the people living in an area.

We must consider the possibility that the development of hospitals in a region may raise the economic value of a community and push out historically underserved populations who can no longer afford to live there. Consequently, the racial and ethnic demographics of the community sharing a zip code with a hospital may be falsely skewed to underreport the community's original makeup of underserved populations. Historically marginalized populations may have been further marginalized out of our analysis. While the data may reflect current gaps, they may dismiss the still-relevant gaps in health equity that are rooted in the past.

## Conclusions and Implications for Health Equity

This article reveals significant differences between where health care leaders live and where they work. More research is needed to investigate the specific impact of these residential disparities and the consequences of potential remedies. Future review should extend to other hospitals beyond those considered “best” or “largest” to investigate the extent of this phenomenon and should examine financial disparities between the incomes of health care leaders, their employees, and the patients they serve. Such research is likely to prove challenging, as techniques exist for masking income through benefits, housing, and incentives from other subsidiary corporations.

The implications of our findings are far-reaching. Contemporary conversations about health equity and addressing gaps in clinical care, health outcomes, and trust between health care systems and marginalized communities are lacking without keen attention to the structural forces that exacerbate such inequities. Potential solutions dovetail nicely with existing conversations about representation of diverse voices in leadership, and understanding such residential gaps is a necessary first step in making that necessary progress.

Health systems select their leadership based on a multitude of factors; perhaps commitment to and experience with the local patient community should be the selection criteria. By no means are we endorsing legal requirements or dictating where any leader lives; rather, perhaps recognizing leaders who come from and share experiences with populations served may prove beneficial for optics, community relationships, and furthering the hospital's mission. Improving the capacity for health care leadership to understand the needs, interests, and lived experiences of their hospital's local community, such as through residential choices, may hold the key to starting the process of healing the communities which health care has historically oppressed.

In 1968, the Kerner Commission—a commission established by former President Lyndon B. Johnson to study the causes of racial unrest—ominously predicted that: “Our nation is moving toward two societies, one black, one white—separate and unequal.”^27^ More than half a century later, the divisions that commission described still plague American health care and pervade nearly every aspect of American society. Nowhere is this clearer than in the residential choices of the nation's health care leaders who, as the above data show, have largely chosen to gate themselves off from the communities that their institutions serve.

The historical contexts and systemic racial and socioeconomic divisions in this country run far deeper than any one hospital executive or dean's choice of residence. However, these choices reflect and reinforce these disparities.

The impacts of such exclusive practices on understanding one another and solidarity cannot be understated. That is not to suggest that the problem presents an easy solution: A rapid large-scale relocation of senior medical professionals to the neighborhoods of their hospitals might create a gentrification crisis, raising rents and displacing the very individuals who are already victims of inequity. However, at a minimum, more candor regarding these residential differences might improve dialogue.

Disparities in health care will not topple through statements and pledges, no matter how heartening nor how long overdue. As in all other aspects of civil life, being present matters: One cannot help wondering how public services might improve if senior health care executives walked the same sidewalks, rode the same buses, sent their children to the same schools, received their health care at the same clinics, and looked out their windows at the same streets as the communities who face entrenched inequities.

## Supplementary Material

Supplemental data

Supplemental data

## References

[1] Braveman P, Gottlieb L. The Social Determinants of Health: it's time to consider the causes of the causes. Public Health Reports. 2014;129:19–31. 10.1177/00333549141291S206 PMC3863696 24385661

[2] Evans MK. Health equity—are we finally on the edge of a new frontier? N Engl J Med. 2020;383:997–999. 32905670 10.1056/NEJMp2005944

[3] Pollack R. Statement on George Floyd's Death and Unrest in America. American Hospital Association. Published June 1, 2020. Available at www.aha.org/press-releases/2020-06-01-statement-george-floyds-death-and-unrest-america Accessed January–December 13, 2020.

[4] Skorton DJ. AAMC Statement on Police Brutality and Racism in America and Their Impact on Health. Association of American Medical Colleges. Published June 1, 2020. Available at https://www.aamc.org/news-insights/press-releases/aamc-statement-police-brutality-and-racism-america-and-their-impact-health Accessed December 13, 2020.

[5] Brooks OT, Peery M. NMA Calls for Open Dialogue on Police Brutality. National Medical Association. Published June 2, 2020. Available at https://www.nmanet.org/news/510933/NMA-Calls-for-Open-Dialogue-on-Police-Brutality.htm Accessed December 18, 2020.

[6] Institute of Medicine (US). How Far Have We Come in Reducing Health Disparities? Progress Since 2000: Workshop Summary. Washington, DC: National Academies Press, 2012. 23193624

[7] U.S. Census Bureau. Births, Deaths, Marriages, and Divorces. Statistical Abstract of the United States: 2011. Published 2011. Available at https://www2.census.gov/library/publications/2010/compendia/statab/130ed/tables/11s0103.pdf Accessed December 22, 2020.

[8] Livingston S. Racism still a problem in healthcare's C-suite. Modern Healthcare. Published February 24, 2018. Available at https://www.modernhealthcare.com/article/20180224/NEWS/180229948/racism-still-a-problem-in-healthcare-s-c-suite Accessed December 22, 2020.

[9] Brownlee D. Why are black male doctors still so scarce in America? Forbes. Published August 11, 2020. Available at https://www.forbes.com/sites/danabrownlee/2020/08/11/why-are-black-male-doctors-still-so-scarce-in-america/?sh=47473d27c2ef Accessed December 22, 2020.

[10] Vega WA, Rodriguez MA, Gruskin E. Health disparities in the Latino population. Epidemiol Rev. 2009;31:99–112. 19713270 10.1093/epirev/mxp008 PMC5044865

[11] Velasco-Mondragon E, Jimenez A, Palladino-Davis AG, et al. Hispanic health in the USA: a scoping review of the literature. Public Health Rev. 2016;37:31. 29450072 10.1186/s40985-016-0043-2 PMC5809877

[12] Eligon J, Nolan K. When police don't live in the city they serve. *New York Times.* August 18, 2016. Available at https://www.nytimes.com/2016/08/19/us/when-police-dont-live-in-the-city-they-serve.html Accessed December 22, 2020.

[13] Muschick P. After we fix gerrymandering, let's make reps live in their districts. The Morning Call. January 30, 2018. Available at https://www.mcall.com/opinion/mc-opi-congress-residency-requirements-gerrymandering-redistricting-muschick-20180124-story.html Accessed December 22, 2020.

[14] Smith DC. Police attitudes and performance: the impact of residency. Urban Aff Q. 1980;15:317–334.

[15] *Becker's Hospital Review* Staff. 100 of the largest hospitals and health systems in America | 2020. Beckers Hospital Review. Published December 22, 2020. Available at https://www.beckershospitalreview.com/lists/100-of-the-largest-hospitals-and-health-systems-in-america-2020.html Accessed July 28, 2020.

[16] *U.S. News & World Report* Staff. Hospital Rankings and Ratings. U.S. News & World Report. Published 2021. Available at https://health.usnews.com/best-hospitals Accessed July 28, 2020.

[17] U.S. Census Bureau. About the American Community Survey. U.S. Census Bureau. Published January 4, 2021. Available at https://www.census.gov/programs-surveys/acs/about.html Accessed January 6, 2021.

[18] U.S. Census Bureau. American Community Survey 5-Year Data (2009–2019). U.S. Census Bureau. Published December 10, 2020. Available at https://www.census.gov/data/developers/data-sets/acs-5year.html Accessed December 10, 2020.

[19] U.S. Census Bureau. American Community Survey 5-Year Data (2015–2019). U.S. Census Bureau. Published December 10, 2020. Available at https://www.census.gov/newsroom/press-releases/2020/acs-5-year.html Accessed December 10, 2020.

[20] Bulatao RA, Anderson NB. Understanding racial and ethnic differences in health in late life: a research agenda. PsycEXTRA Dataset. 2004. 20669466

[21] SAS/STAT^®^, SAS Institute Inc., NC, USA.

[22] Kochhar R, Taylor P, Fry R. Wealth Gaps Rise to Record Highs Between Whites, Blacks and Hispanics. Washington, DC: Pew Research Center. Published July 26, 2011. Available at https://www.pewresearch.org/social-trends/2011/07/26/wealth-gaps-rise-to-record-highs-between-whites-blacks-hispanics/ Accessed January 21, 2021.

[23] Rosenbaum S, Kindig DA, Bao J, et al. The value of the nonprofit hospital tax exemption was $24.6 billion in 2011. Health Aff (Millwood). 2015;34:1225–1233. 26085486 10.1377/hlthaff.2014.1424 PMC7153767

[24] Herring B, Gaskin D, Zare H, et al. Comparing the value of nonprofit hospitals' tax exemption to their community benefits. INQUIRY J Health Care Organ Prov Financ. 2018;55:004695801775197. 10.1177/0046958017751970 PMC5813653 29436247

[25] Health Resources and Services Administration. MUA Find. HRSA. Available at https://data.hrsa.gov/tools/shortage-area/mua-find Accessed January 6, 2021.

[26] Bhavsar NA, Kumar M, Richman L. Defining gentrification for epidemiologic research: a systematic review. PLoS One. 2020;15:e0233361. 32437388 10.1371/journal.pone.0233361 PMC7241805

[27] Kerner O. The Kerner Report: The National Advisory Commission on Civil Disorders. New York: Pantheon, 1988.

