# Supplementary material for: Do Hospital Leaders Live in the Communities They Serve? A Comparative Analysis
Source: Health Equity. 2022 Apr 21;6(1):313–21. doi: 10.1089/heq.2021.0147 (PMC9081031; doi:10.1089/heq.2021.0147)

**Supplemental Figure 2: *Distribution of* *Hispanic & Latinx Populations in Hospital and Leadership Zip Codes***


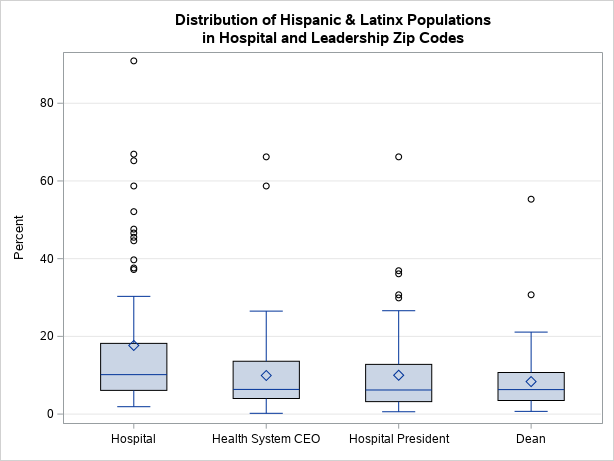

Supplement: Supplemental data [file Suppl_FigureS2.docx]
